# When Constriction Is Not Just the Pericardium: Extrinsic Cardiac Encasement by Thymic Squamous Cell Carcinoma

**DOI:** 10.7759/cureus.105678

**Published:** 2026-03-23

**Authors:** Waleed Dawelbeit, Z Al-Hashami, Marwa Makhloof, Noof Alkharoosi, Ahmed Basuoni

**Affiliations:** 1 Cardio-oncology, Sultan Qaboos Comprehensive Cancer Care and Research Center, Muscat, OMN; 2 Medical Oncology, Sultan Qaboos Comprehensive Cancer Care and Research Center, Muscat, OMN; 3 Cardiology Clinical Physiology, Sultan Qaboos Comprehensive Cancer Care and Research Center, Muscat, OMN

**Keywords:** cardiac encasement, constrictive pericarditis, malignant pseudo-constriction, pericardial tamponde, pericarditis, thymic squamous cell carcinoma, ventricular interdependence

## Abstract

Constrictive pericarditis (CP) typically primarily involves pericardial pathology, but mediastinal malignancies can create an identical hemodynamic profile through direct extrinsic encasement. We report a 58-year-old male patient with metastatic thymic squamous cell carcinoma presenting with refractory right-sided heart failure and anasarca. Although his history initially suggested radiation-induced or post-COVID-19 pericarditis, right heart catheterization and echocardiography revealed classic constrictive physiology, including a “septal bounce” and ventricular interdependence with a preserved ejection fraction. A four-month trial of corticosteroids and colchicine failed to improve his hemodynamics. Positron emission tomography-computed tomography (PET/CT) subsequently confirmed that the thymic mass had directly infiltrated the mediastinal pleura and pericardium, creating a rigid shell encasing the heart and compressing the inferior vena cava. Due to the tumor's surgical inaccessibility and the failure of medical treatment, the patient was transitioned to palliative care. This case of “malignant pseudo-constriction” emphasizes the diagnostic challenge of distinguishing reversible inflammatory pericarditis from irreversible malignant encasement. In patients with advanced mediastinal tumors, persistent constrictive symptoms despite anti-inflammatory therapy should raise high clinical suspicion for direct tumor infiltration. This condition, which can be diagnosed utilizing advanced imaging modalities, carries a poor prognosis and typically precludes surgical intervention.

## Introduction

Thymic squamous cell carcinoma (SCC) is a rare and aggressive epithelial malignancy of the mediastinum, often characterized by local invasion of vital thoracic structures [1]. While cardiac complications in oncology patients are frequently attributed to treatment-induced cardiotoxicity or malignant pericardial effusion, the development of constrictive physiology due to direct tumor encasement is an underreported phenomenon. Constrictive pericarditis (CP) typically involves the stiffening and scarring of the pericardial layers, but “pseudo-constriction” can occur when an extrinsic mass creates a non-compliant shell around the myocardium, restricting diastolic filling and inducing ventricular interdependence [2-6]. 

In patients with multiple causative factors “hits”- including thoracic radiation therapy (RTx), previous malignancies, and viral insults such as COVID-19 - distinguishing between inflammatory pericarditis and mechanical tumoral encasement is clinically challenging. Traditional anti-inflammatory therapies, such as corticosteroids and colchicine, are the cornerstone for managing transient or chronic pericarditis [7]; however, they are largely ineffective when the restrictive driver is a solid malignant mass. Understanding the mechanical nature of this obstruction is critical for determining surgical candidacy and prognostic expectations.

## Case presentation

A 58-year-old male patient was diagnosed in April 2016 with metastatic SCC of thymic origin, featuring pleural and paraspinal metastases. He received palliative chemotherapy with carboplatin and gemcitabine until 2018; however, this was discontinued due to poor tolerance. He subsequently received palliative radiotherapy (RTx) directed at the mediastinal and paraspinal masses. His clinical course was complicated in early 2022 by severe COVID-19 pneumonia and associated pericarditis and pericardial effusion, which progressed to pericardial tamponade necessitating emergent pericardiocentesis. Subsequently, the patient developed radiation-induced superior vena cava (SVC) fibrosis, which was managed with stenting and re-stenting in late 2023 to maintain venous patency.

**Figure 1:**
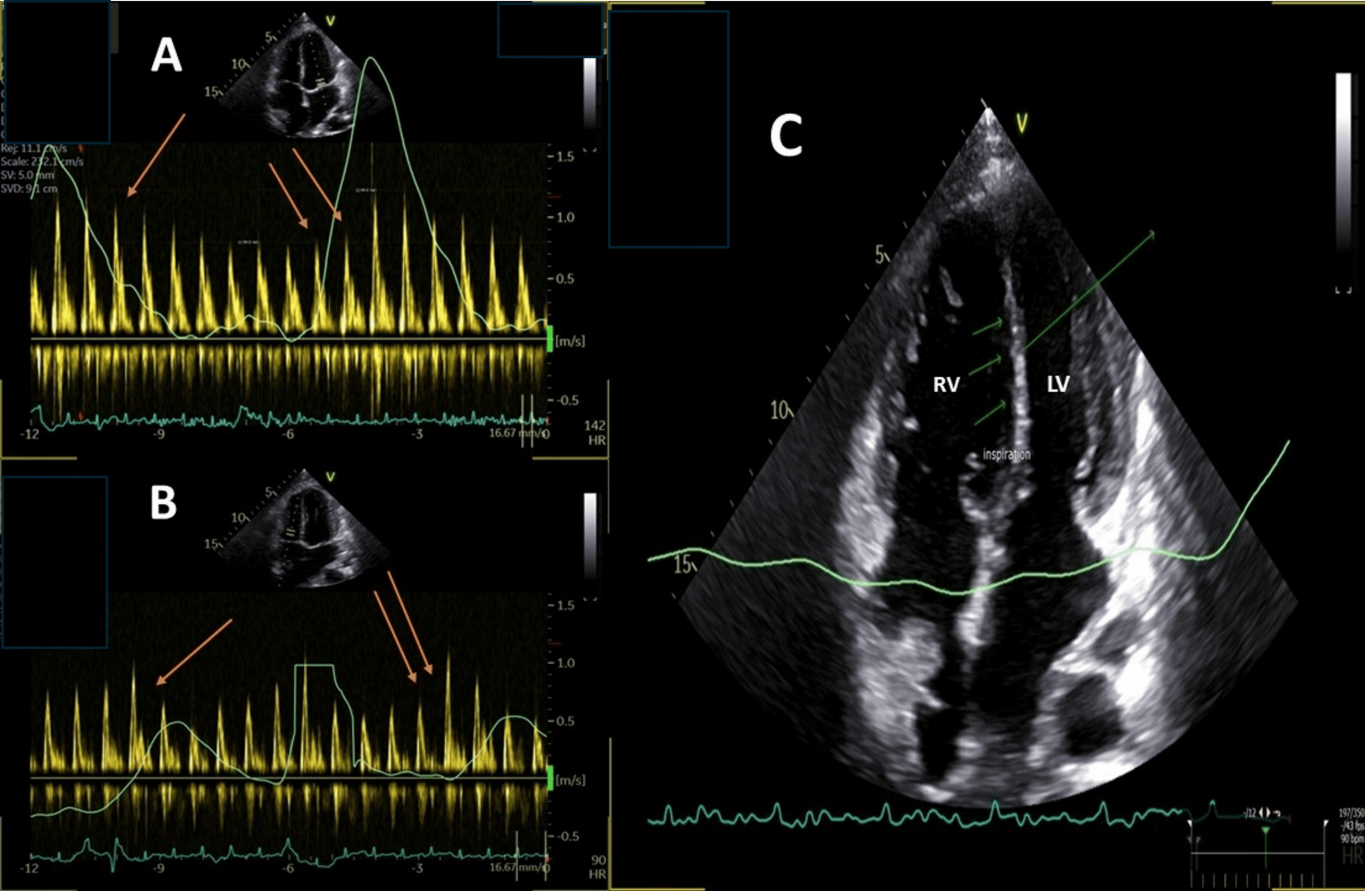
Transthoracic echocardiographic assessment of ventricular interdependence Transthoracic echocardiography illustrates the hallmark hemodynamic findings of constrictive physiology. Panel A: Pulsed-wave Doppler of transmitral inflow shows a significant reduction in velocity during inspiration (single red arrow) and a reciprocal increase during expiration (double red arrows). Panel B: Pulsed-wave Doppler of transtricuspid inflow demonstrates a reduction in velocity during expiration (single red arrow) and a marked increase during inspiration (double red arrows). Panel C: The apical four-chamber view identifies the interventricular septum (single green arrow) with a prominent "septal bounce" (triple green arrows), diagnostic of reciprocal filling within a fixed, non-compliant cardiac volume.

In July 2023, the patient presented with worsening anasarca and progressive exertional dyspnea; his refractory volume overload prompted the initial diagnostic workup. Echocardiography revealed significant respiratory variation in mitral inflow and a prominent ventricular septal bounce, with a plethoric inferior vena cava (IVC), findings highly suggestive of ventricular interdependence and constrictive physiology (Figure 1, Videos 1, 2)

**Video 1:** Transthoracic echocardiographic assessment of respiratory phasic variation and ventricular interdependence This looped video illustrates the mechanical "rigid shell" effect. Upper panels: The apical four-chamber view identifies the interventricular septum (single green arrow, upper-left) exhibiting a paradoxical "septal bounce" toward the left ventricle during inspiration, reflecting space competition within the non-compliant mediastinum. Lower panels: Integrated pulsed-wave Doppler demonstrates exaggerated respiratory phasic variation. Transmitral inflow (lower-left) shows a marked reduction in peak E-wave velocity during inspiration, while transtricuspid inflow (lower-right) shows a reciprocal increase, confirming restrictive physiology from extrinsic encasement.

**Video 2:** Transthoracic echocardiography demonstrating ventricular interdependence Apical four-chamber view, acquired at a depth of 16 cm, illustrates the mechanical consequences of extrinsic cardiac encasement. The single green arrow identifies the interventricular septum, which exhibits a characteristic "septal bounce"—a rapid, paradoxical shift during the respiratory cycle. This displacement occurs as the ventricles compete for limited filling space within the rigid mediastinal shell (ventricular interdependence).

To further investigate these findings, cardiac magnetic resonance imaging (MRI) and right heart catheterization (RHC) were performed in August 2023, which confirmed a hemodynamic profile consistent with a constrictive physiology. However, the etiology remained ambiguous, given the patient's history of radiation, viral COVID-19 infection, and active malignancy. Consequently, a four-month trial of high-dose corticosteroids and colchicine was initiated to address potential post-viral or radiation-induced inflammatory pericarditis.

**Figure 2:**
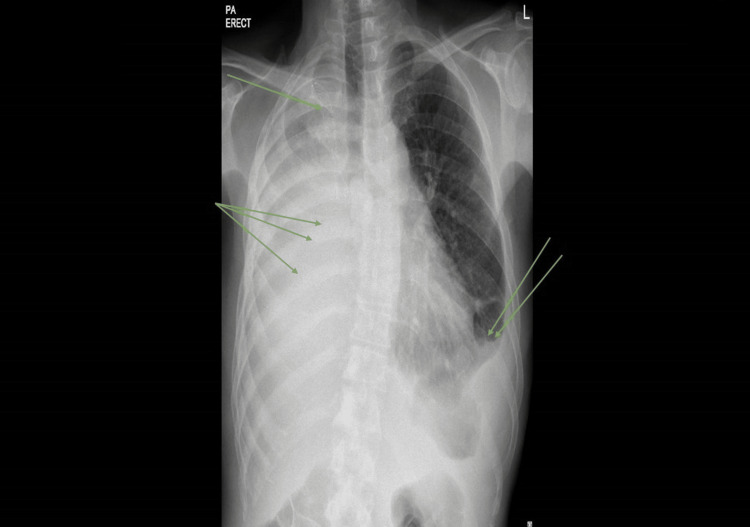
Posteroanterior (PA) erect chest radiograph The PA chest X-ray demonstrates significant opacification of the right hemithorax, indicative of the primary mediastinal mass. The left lung remains clear without evidence of consolidation, though a mild left-sided pleural effusion is present. The single green arrow identifies the metallic stent within the superior vena cava (SVC), previously placed to manage radiation-induced fibrosis. The double green arrows highlight the left-sided pleural effusion, and the triple green arrows delineate the extensive right-sided mass responsible for the hemithorax opacification and extrinsic cardiac encasement.

**Figure 3:**
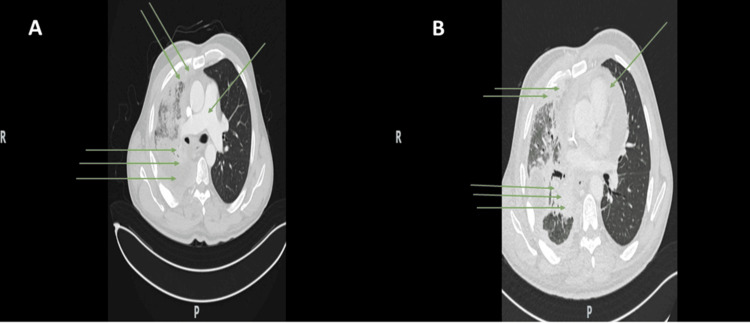
Contrast-enhanced chest computed tomography (CT) Axial contrast-enhanced CT images at the level of the great vessels (A) and upper cardiac chambers (B) demonstrate significant disease progression and malignant cardiac encasement. The mass directly infiltrates the mediastinal pleura and pericardium, becoming inseparable from the adjacent epicardial fat pads. Panel A: The single green arrow identifies the pulmonary artery and its primary branches. Double green arrows highlight the enhancing malignant soft tissue in the right anterior mediastinum. Triple green arrows demonstrate the paravertebral soft tissue involvement adjacent to the thoracic spine, encroaching upon the adjacent right-sided neuroforamina and intercostal musculature. Panel B: The single green arrow points to a moderate pericardial effusion within the "rigid shell." The double green arrows identify the right-sided anterior mediastinal soft tissue. The triple green arrows delineate the diffuse, nodular enhancing pleural thickening along the right lung, as well as the paraspinal mass responsible for the extrinsic compression of the inferior vena cava (IVC) and deformity of the right atrium (RA).

Despite adherence to the anti-inflammatory regimen, the patient’s condition failed to improve. By June 2024, the patient was admitted to the intensive care unit (ICU) with hypoxic respiratory failure. Imaging, including positron emission tomography-computed tomography (PET/CT), demonstrated that the mediastinal mass had progressed significantly, with direct infiltration of the mediastinal pleura and pericardium surrounding the heart. This created a “rigid shell” that encased the major mediastinal blood vessels and the heart. The mass further caused a physical deformity of the right atrium (RA) and moderate extrinsic compression of the IVC. (Figures 2, 3 and Video 3).

**Video 3:** Transthoracic echocardiography demonstrating malignant cardiac encasement and subsequent ventricular interdependence This looped apical four-chamber view, acquired at a depth of 26 cm, provides a wide-field visualization of the malignant "rigid shell." A large, solid mass is identified posterior to the atria, while a prominent, echogenic layer of malignant infiltration is seen circumferentially encasing the heart. This extrinsic sheath acts as a non-compliant cage, restricting diastolic filling and driving the pathological ventricular interdependence.

Despite aggressive intravenous (IV) diuretics, the patient developed refractory diuretic resistance and acute kidney injury (AKI). A follow-up echocardiogram in December 2024 showed a preserved ejection fraction (EF 68%) but a persistent severe ventricular septal bounce (Videos 4, 5).

**Video 4:** Apical three-chamber view of ventricular interdependence and posterior left ventricular wall encasement This looped transthoracic echocardiogram utilizes an apical three-chamber view to demonstrate the mechanical restriction of the left ventricle. A prominent septal bounce and clear ventricular interdependence are visible, indicative of the constrained intracardiac volume. The posterior wall is bordered by a distinct, echogenic thick layer of malignant infiltration that circumferentially surrounds the heart, creating the physiological "rigid shell." The green bidirectional arrows specifically highlight this infiltrating tissue, which serves as the primary driver of the patient's constrictive hemodynamics.

**Video 5:** Apical biplane imaging demonstrating ventricular interdependence This apical biplane view (Left: four-chamber; Right: modified two-chamber) provides a simultaneous multi-plane assessment of the cardiac chambers. The video captures the "septal bounce" and pronounced ventricular interdependence in real-time. This illustrates how the extrinsic "rigid shell" restricts global ventricular filling, forcing a reciprocal septal shift as the ventricles compete for space during the respiratory cycle.

Given the extensive, inoperable infiltration and failure of medical optimization strategies, the patient was transitioned to the palliative care team for best supportive care. He passed away two weeks later.

## Discussion

The clinical presentation of this patient represents a profound diagnostic and therapeutic paradox, where a metastatic thymic SCC resulted in a state of malignant pseudo-constriction. The diagnostic challenge was amplified by the patient's multifactorial history, presenting a triple cause of potential triggers: radiation-induced fibrosis, post-viral inflammation from COVID-19, and direct malignant infiltration. While echocardiography and RHC confirmed ventricular interdependence and a “dip-and-plateau” hemodynamic signature, these features are physiologically non-specific [8,9]. They confirm the physiology but not the cause. In this case, the tumor effectively replaced the pericardium as the heart's limiting boundary, creating a fixed-volume system. The early RTx he received made it difficult to distinguish solid-tissue infiltration from simple fibrotic remodeling, and a biopsy could not be obtained due to the high surgical and interventional risks. This was a major limitation in confirming the definite etiology of the infiltrating tissue.

The therapeutic course of this patient further underscores the management challenges inherent in pseudo-constriction; it also highlights the ineffectiveness of anti-inflammatory medications. In classic or post-viral CP, a trial of corticosteroids and colchicine often yields clinical improvement [10]. However, in this case, the four-month trial provided late-stage clinical evidence supporting the conclusion that the restriction was mechanical rather than inflammatory. Furthermore, the physiology was complicated by dual venous obstruction: SVC occlusion from radiation-induced fibrosis and direct IVC compression by the tumor mass. This rendered standard heart failure management ineffective, as the venous congestion remained refractory to diuresis, precipitating anasarca and cardio-renal syndrome.

The most critical distinction lies in surgical candidacy. In classic CP, a pericardiectomy is often curative. However, malignant encasement involves a “strangling” physiology that encases the heart, infiltrates the mediastinal pleura, and the great vessels. The absence of a clear surgical option rendered the condition surgically unresectable. Advanced multimodal imaging, specifically PET/CT, remains the gold standard for identifying the anatomical driver of such restrictive physiology and guiding the transition to palliative care.

## Conclusions

In advanced mediastinal malignancies, clinicians must look beyond the pericardial sac when evaluating constrictive physiology. This case demonstrates that “malignant pseudo-constriction” from direct tumor encasement can replicate the hemodynamic profile of a classic constrictive pericarditis. Differentiating this mechanical obstruction from inflammatory or radiation-induced pericarditis is critical, as malignant encasement renders anti-inflammatory therapy ineffective and precludes surgical intervention. Recognizing direct infiltration through multimodal imaging is essential for early prognostic accuracy and timely alignment of palliative care goals. 
